# Interactions among genes in the ErbB-Neuregulin signalling network are associated with increased susceptibility to schizophrenia

**DOI:** 10.1186/1744-9081-3-31

**Published:** 2007-06-28

**Authors:** Isabel Benzel, Aruna Bansal, Brian L Browning, Nicholas W Galwey, Peter R Maycox, Ralph McGinnis, Devi Smart, David St Clair, Phillip Yates, Ian Purvis

**Affiliations:** 1 Psychiatry CEDD, GlaxoSmithKline, New Frontiers Science Park, Third Avenue, Harlow, Essex, CM19 5AW Harlow, Essex, UK; 2 Discovery and Pipeline Genetics, GlaxoSmithKline, Harlow, Essex, UK; 3 Discovery and Pipeline Genetics, GlaxoSmithKline, Research Triangle Park, North Carolina, USA; 4 Department of Mental Health, University of Aberdeen, Institute of Medical Sciences, Aberdeen AB25 2ZD, UK; 5 Scottish National Blood Transfusion Service, Aberdeen AB25 2ZW, UK; 6 Therapeutic Area Team, GlaxoSmithKline, Stevenage, Hertfordshire, UK; 7 Department of Nutrition, The University of Auckland, Private Bag 92019, Auckland, New Zealand; 8 Wellcome Trust Sanger Institute, Cambridgeshire, CB10 1SA, UK

## Abstract

**Background:**

Evidence of genetic association between the NRG1 (Neuregulin-1) gene and schizophrenia is now well-documented. Furthermore, several recent reports suggest association between schizophrenia and single-nucleotide polymorphisms (SNPs) in ERBB4, one of the receptors for Neuregulin-1. In this study, we have extended the previously published associations by investigating the involvement of all eight genes from the ERBB and NRG families for association with schizophrenia.

**Methods:**

Eight genes from the ERBB and NRG families were tested for association to schizophrenia using a collection of 396 cases and 1,342 blood bank controls ascertained from Aberdeen, UK. A total of 365 SNPs were tested. Association testing of both alleles and genotypes was carried out using the fast Fisher's Exact Test (FET). To understand better the nature of the associations, all pairs of SNPs separated by ≥ 0.5 cM with at least nominal evidence of association (*P *< 0.10) were tested for evidence of pairwise interaction by logistic regression analysis.

**Results:**

42 out of 365 tested SNPs in the eight genes from the ERBB and NRG gene families were significantly associated with schizophrenia (*P *< 0.05). Associated SNPs were located in ERBB4 and NRG1, confirming earlier reports. However, novel associations were also seen in NRG2, NRG3 and EGFR. In pairwise interaction tests, clear evidence of gene-gene interaction was detected for NRG1-NRG2, NRG1-NRG3 and EGFR-NRG2, and suggestive evidence was also seen for ERBB4-NRG1, ERBB4-NRG2, ERBB4-NRG3 and ERBB4-ERBB2. Evidence of intragenic interaction was seen for SNPs in ERBB4.

**Conclusion:**

These new findings suggest that observed associations between NRG1 and schizophrenia may be mediated through functional interaction not just with ERBB4, but with other members of the NRG and ERBB families. There is evidence that genetic interaction among these loci may increase susceptibility to schizophrenia.

## Background

Schizophrenia is a complex psychiatric disorder which affects 0.5–1% of the world wide adult population. A number of putative schizophrenia susceptibility genes have been identified recently [1,2]. Genomewide linkage analysis on large Icelandic pedigrees showed evidence for linkage with an initial LOD score of 3 on chromosome 8p13, which led to the identification of the Neuregulin-1 gene (NRG1) as a potential genetic risk factor (O.R. = 2) for schizophrenia [3]. The association was confirmed in a large Scottish case control data set [4], and both studies indicated a core haplotype (HAP_ICE_) as the DNA variation carrying most of the risk for schizophrenia. Subsequently, 15 published studies in schizophrenia data sets of various Asian and Caucasian ethnic backgrounds have detected association in NRG1 SNPs or haplotypes, while only four studies were not able to replicate the association (see [5] and [6] for a comprehensive review). The association studies reported thus far strongly suggest a true effect of NRG1 as a susceptibility gene for schizophrenia. However, some caution should be exercised before calling NRG1 a true "schizophrenia gene" due to the lack of a known functional effect of the identified NRG1 variants, the allelic heterogeneity reported across several studies and the multiple SNPs and haplotypes analysed in different studies. Nonetheless, several recent publications report an association between schizophrenia and ErbB4, one of the receptors for NRG1 [7-9]. This provides indirect evidence supporting NRG1 association with schizophrenia and a role for aberrant neuregulin signalling in this disorder.

The Neuregulin-ErbB signalling network is involved in a multitude of processes in the developing and adult brain. Neuregulins for example promote neuronal migration and differentiation, regulate the expression of neurotransmitter receptors, influence glial proliferation, survival and differentiation and play a role in synaptic plasticity.

The neuregulins (NRG) are cell-cell signalling proteins that are ligands for receptor tyrosine kinases of the ErbB family. Whereas NRG1 is known to play essential roles in nervous system and heart development, as well as in the adult brain (see above), less is known about the other members of the NRG gene family, NRG2, -3 and -4 [10,11].

**Figure 1:**
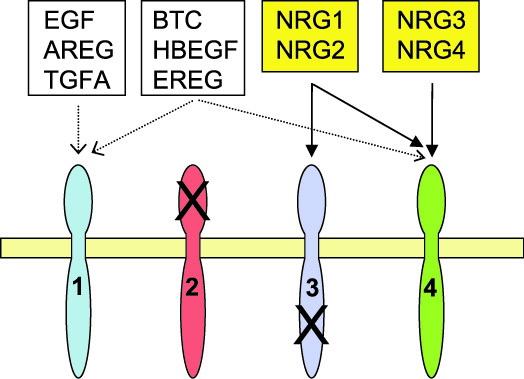
**Binding specificities of ErbB receptor family ligands**. EGF (epidermal growth factor), AREG (amphiregulin) and TGFA (transforming growth factor-α) bind EGFR (ErbB1) only. BTC (Betacellulin), HBEGF (heparin-binding EGF-like growth factor) and EREG (Epiregulin) bind EGFR and ErbB4. NRG1 and NRG2 bind ErbB3 and ErbB4. NRG3 and NRG4 are specific for ErbB4. Receptors form homo- and heterodimers after ligand-binding (not shown). As indicated (X), ErbB2 has no ligand-binding capacity and ErbB3 has no active kinase domain. Only NRG1-4 and ERBB1-4 genes were investigated in this study. (Modified from [46]).

The ErbB family comprises four homologous typeI receptor tyrosine kinases (RTKs). The EGFR (epidermal growth factor receptor, HUGO name for ErbB1), ErbB3 and ErbB4 receptors can bind ligands, whereas ErbB2 lacks a ligand binding domain and functions as a preferred and very potent co-receptor. ErbB3 is devoid of an active kinase domain. ErbB4 is the only ErbB family member that binds all four neuregulins, as well as several proteins that had originally been identified as EGFR ligands (Fig. 1). Furthermore, NRG1 and NRG2 can also bind to ErbB3. In contrast, none of the neuregulins bind to EGFR which instead binds unrelated ligands such as EGF, amphiregulin and others (see Fig. 1).

Ligand binding to the extracellular domain of ErbB family members leads to receptor homo-and heterodimerisation and activation of various intracellular signalling pathways such as the Ras-Raf-MAPK and the PI-3 Kinase pathways (reviewed in [12]).

In order to investigate the involvement of neuregulin pathway genes in schizophrenia, beyond the previously published association with NRG1 and ERBB4, we have tested all eight genes from the ERBB and NRG families for association with schizophrenia. We have investigated both single genes and gene-gene interactions, using a collection of schizophrenia cases and blood bank controls from Aberdeen, Scotland.

## Methods

### Subjects

396 Caucasian cases were ascertained in Aberdeen, Scotland. All have a basic diagnosis of schizophrenia or schizoaffective disorder according to the Operational Criteria Checklist (OPKRIT). 285 of the cases were male; 111 were female. The mean recruitment age was 44 yr with a standard deviation of 14 yr.

1342 Controls were ascertained as a series of anonymous blood donors from Aberdeen, Scotland. Due to subject anonymity, characteristics such as schizophrenia- status, age and ethnic group could not be determined. However at the time of sample collection, the Aberdeen Blood Transfusion Service made a concerted effort to exclude any non-Caucasian samples. At the time of this study, non-Caucasians constituted around 2% of the Aberdeen population but a lower percentage of Aberdeen blood donors. 724 control subjects were male; 618 were female. Informed consent was obtained for all subjects involved in this study.

As calculated by the power equations of Risch and Teng [13], under a multiplicative model of association, a sample of 400 cases and 1300 controls has 80% power to detect disease association at an α = 0.05 significance level for SNPs having a genotypic relative risk of 1.5 [14] and susceptibility allele frequencies between 0.07 and 0.9.

### Polymorphism selection and genotyping

SNPs from each gene were selected at an approximate spacing of 1 SNP every 10 kb. Six of these SNPs are located in potentially functional regions of the genes, five in exons and one in a promoter region (Table 1). Among the SNPs that are in exons, two are synonymous, but the other three produce amino acid changes. The size of these genes varied greatly, from 23 kb to 1110 kb, and there was a corresponding variation in the number of SNPs analysed, from 4 to 109 (for further details see Table 2, in the Results section).

**Table 1:** SNPs in potentially functional gene regions included in the present study.

**Gene**	**Polymorphism**^1^	**Chromosome**	**Position**^2^	**Region**	**Base change**	**Amino acid change**
EGFR	rs2072454	7	54988557	Exon 4	C/T	Asn/Asn
ERBB2	rs1801200	17	35133114	Exon 20	A/G	Val/Ile
ERBB2	rs1058808	17	35137563	Exon 30	C/G	Pro/Ala
ERBB4	rs3748962	2	212077370	Exon 27	C/T	Val/Val
NRG1	rs6994992	8	31615123	Promoter	C/T	-
NRG1	rs3924999	8	32572900	Exon 2	C/T	Gln/Arg

^1^Polymorphism names are taken from the dbSNP website, [47].

^2^From map NCBI 35.

**Table 2:** Polymorphism in genes in the families NRG and ErbB investigated in this study.

**Gene**	**Chromosome**	**Size (kb)**	**No. of analysable SNPs**	**No. of SNPs with p < 0.10**^1^	**No. of SNPs with p < 0.05**^1^
NRG1	8	1120	94	22 (23%)	11 (12%)
NRG2	5	196	16	2 (13%)	1 (6%)
NRG3	10	1110	105	21 (20%)	10 (10%)
NRG4	15	70	9	0	0
EGFR	7	188	22	7 (32%)	6 (27%)
ERBB2	17	29	4	1 (25%)	0
ERBB3	12	23	6	0	0
ERBB4	2	1160	109	26 (24%)	14 (13%)

^1 ^Either genotypic test or allelic test of association.

Genotyping was performed using a modification of the single base chain extension assay previously described [15], at GlaxoSmithKline, UK. A SNP passed QC if over 80% of genotype calls were successful.

### Statistical analysis

All SNPs were tested for departure from Hardy Weinberg equilibrium (HWE) in controls using a chi squared test with one degree of freedom. This test compares the expected genotype frequencies calculated from the allele frequencies, with observed genotype frequencies. A departure from HWE in controls may indicate errors in the data.

Association testing of both alleles and genotypes was carried out using the 'PROC FREQ' fast Fisher's Exact Test (FET) procedure in the statistical software package SASv8.2 (SAS Institute Inc., Cary, NC, USA). The Fast Fisher's Exact Test computes exact *P*-values for general contingency tables using the network algorithm developed by [16]. The network algorithm provides substantial advantages over direct enumeration and is rapid and accurate. The exact test is used in preference to the asymptotic chi-square *P*-value as it is more accurate at loci where the minor allele frequency is low.

In order to model and test for interactions between SNPs in different candidate genes, standard logistic regression analysis was performed using the statistical software package R (The R Project for Statistical Computing, [17]). Following Cordell [18] the model tested had the following form:

*log(p/(1-p)) *= *μ *+ *a*_1 _*x*_1 _+ *d*_1 _*z*_1 _+ *a*_2 _*x*_2 _+ *d*_2 _*z*_2 _+ *i*_*aa *_*x*_1 _*x*_2 _+ *i*_*ad *_*x*_1 _*z*_2 _+ *i*_*da *_*z*_1 _*x*_2 _+ *i*_*dd *_*z*_1 _*z*_2_

where *x*_1_, *x*_2_, *z*_1 _and *z*_2 _are variables determined by the genotype under consideration, and *μ*, *a*_1_, *a*_2_, *d*_1_, *d*_2_, *i*_*aa*_, *i*_*ad*_, *i*_*da *_and *i*_*dd *_are parameters to be estimated. Here, genotypes at two SNPs are being modelled and, for each of the 9 possible genotype combinations at the two SNPs, *p *is the proportion of subjects with the genotype who are cases and *(1-p) *is the proportion of subjects with the genotype who are controls. The first five terms in the equation represent the effects of the two SNPs on *log(p/(1-p)) *if there is no statistical interactions between the two SNPs. A non-zero value for one or more of the remaining four coefficients (*i*_*aa*_, *i*_*ad*_, *i*_*da*_, *i*_*dd*_) indicates the presence of interaction. Statistical significance was assessed by a likelihood ratio test, comparing the full model above with a reduced model in which the last four terms are absent (i.e. in which the coefficients *i*_*aa*_, *i*_*ad*_, *i*_*da *_and *i*_*dd *_are all set to zero).

A significant interaction effect in the logistic regression analysis indicates that the combined effect of the two SNPs is significantly different from the sum of their individual effects. Pair-wise interactions may be of three types, namely 'and', 'or' and 'exclusive or', defined as follows. In an 'and' relationship, the effect of having the risk-enhancing genotypes at both SNP loci is larger than the sum of the single-locus effects. In an 'or' relationship, the effect of having the risk-enhancing genotype at both loci is smaller than the sum of the single-locus effects. In the final type of interaction, named 'exclusive or', the effect of having the risk-enhancing genotype at both loci is smaller than that of having a risk-enhancing genotype at only one locus. For each SNP pair, each of these configurations gives rise to a contingency table, classified by genotype group (risk-enhancing or risk-reducing) and phenotype (case or control). These contingency tables were evaluated, and the configuration with the lowest *P*-value for association with the phenotype was identified. This configuration indicated the type of interaction between the two loci in question.

## Results

### Data checking

Although controls were selected from the same geographic area as the cases, a test was conducted to determine the presence of any latent population stratification. The lambda statistic of Devlin et al. [19], calculated on 4,250 SNPs distributed over the whole genome, showed no evidence of stratification (population heterogeneity, overdispersion: Genotypic exact lambda = 1.061, Allelic exact lambda = 1.000), indicating that no genomic-control adjustment is required when performing tests of genotypic and allelic association on these subjects.

Of 374 SNPs submitted for genotyping, 3 were dropped from analysis due to monomorphism in cases and controls, and another 6 were dropped due to departure from Hardy-Weinberg Equilibrium (P < 0.001). Among the remaining SNPs, the proportion of individuals successfully genotyped ranged from 0.804 to 0.99, with a median of 0.97. A subset of the individuals studied was genotyped in duplicate, giving a total of 14,323 duplicate genotypes. Of these, only 2 (= 0.014%) were inconsistent. The 365 SNPs retained, located in 8 genes, were subjected to single-point analysis. The number of SNPs per gene is given in Table 2. The median frequency of the minor allele among the SNPs analysed was 22.9 %, but there were 35 SNPs (9.6% of those analysed) for which the minor allele frequency was less than 5%. These SNPs, selected prior to the availability of the HapMap [20], are less informative than the others; however, as they do provide some additional information they were retained in the analysis.

### Single-point results

Both allelic and genotypic tests of association were conducted. Detailed results are provided in Additional file 1. Significant results (*P *< 0.05) were observed in five genes, namely NRG1, NRG2, NRG3, EGFR and ERBB4. These confirmed previous findings on NRG1 and ERBB4 and suggest possible involvement also of NRG2, NRG3 and EGFR. Borderline evidence of association (*P *< 0.10) was observed for one SNP in ERBB2. Haplotypic tests of association (not shown) provided similar results to the single-point results described here. If none of the 365 single SNPs were associated with the disease status (the null hypothesis), we would expect 18 SNPs to be significant at the 0.05 level and 37 SNPs to be significant at the 0.10 level on an allelic test. For the allelic test there were 34 SNPs significant at the 0.05 level (9.3%) and 59 SNPs significant at the 0.10 level (16.2%). For the genotypic test there were 24 SNPs significant at the 0.05 level (6.6%) and 62 SNPs significant at the 0.10 level (17.0%). This excess of significantly associated SNPs, beyond the numbers expected by chance when accounting for the multiple tests, provides evidence that some of these associations are real.

**Figure 2:**
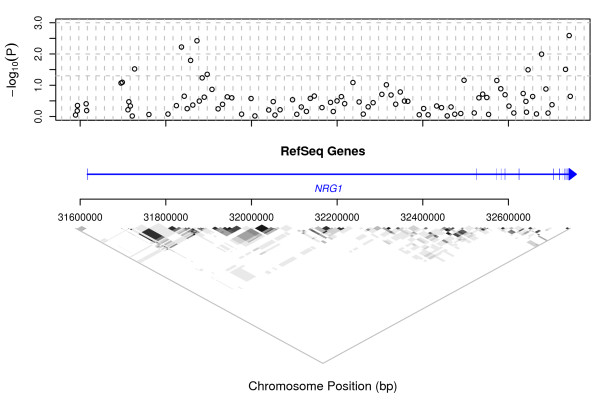
**Genotypic single-point association test results and LD over NRG1**. In the plot of -log_10_(*P*) v. map position, horizontal dashed lines are at *P *= 0.05, 0.01 and 0.001. Vertical dashed lines are at 20 kb intervals. The region covered by the gene is indicated in the centre of the figure by a horizontal line, with an arrow head indicating the direction of transcription. A crossing vertical line indicates the position of each exon. At the base of the figure, LD between SNP loci is indicated by shading. White: *r*^2 ^= 0; black: *r*^2 ^= 1.

The distribution of evidence for single-point association with schizophrenia, and linkage disequilibrium (LD), over gene NRG1 are presented in Fig. 2. In this figure, the magnitude of association of each SNP with schizophrenia is indicated by the log-transformed *P*-value. For the sake of simplicity, only values arising from the genotypic exact test are shown; allelic test results followed a very similar pattern. Across both tests, eleven SNPs in this gene showed significant genotypic association with schizophrenia (*P *< 0.05), and in three instances the association was highly significant (*P *< 0.01) (see Additional file 1: SNP RS776401: map position 031836504 on Chr 8, risk genotype odds ratio (OR) = 1.461, 95% confidence interval (CI) 1.160–1.840; SNP RS1383887: map position 031872698, OR = 1.589, CI 1.213–2.081; SNP RS-GSK8116812: map position 032741837, OR = 1.651, CI 1.242–2.196). A putatively functional SNP in Exon 2, coding a non-synonymous (Gly/Arg) change, showed allelic association to schizophrenia with *P *= 0.0279 (SNP RS3924999: map position 032572900, OR = 1.302, CI 1.028–1.648). It is possible that the cluster of associations observed at the 3' end of the gene, to the right of the figure, has arisen through LD with this non-synonymous change or another underlying functional variant. Further evidence of association is seen at the 5' end of the gene, closer to Exon 1. The latter associations fall beyond the apparent reach of LD.

**Figure 3:**
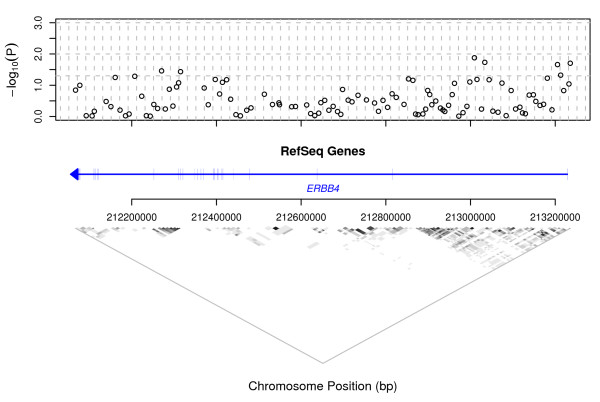
**Genotypic single-point association test results and LD over ERBB4**. In the plot of -log_10_(*P*) v. map position, horizontal dashed lines are at *P *= 0.05, 0.01 and 0.001. Vertical dashed lines are at 20 kb intervals. The region covered by the gene is indicated in the centre of the figure by a horizontal line, with an arrow head indicating the direction of transcription. A crossing vertical line indicates the position of each exon. At the base of the figure, LD between SNP loci is indicated by shading. White: *r*^2 ^= 0; black: *r*^2 ^= 1.

The corresponding information for gene ERBB4 is presented in Fig. 3. In this gene, fourteen SNPs were significantly associated with schizophrenia by either the allelic or genotypic test, including one allelic test result which was highly significant (*P *< 0.01: SNP RS6754674: map position 213033762 on Chr 2, OR = 1.387, CI 1.056–1.822). Again, the graph of genotypic test results shows strong evidence of association occurring most commonly at the two ends of the gene, particularly at the 5'-end, to the right of the figure, although the pattern is not as clear as in the case of NRG1. This region of the gene represents a 0.15 Mb region of moderate LD, so it is possible that a single underlying variant, in LD with all of the SNPs that show significant association, accounts for the observed association in this region.

**Figure 4:**
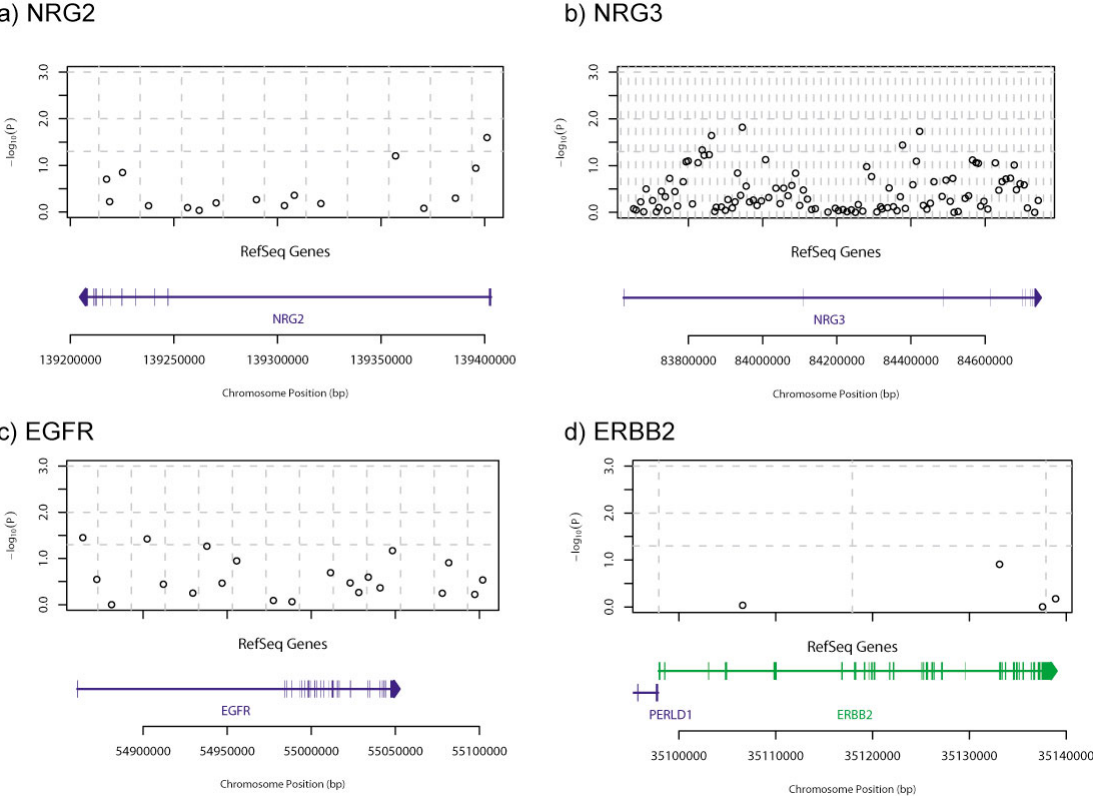
**Genotypic single-point association test results and LD over NRG2, NRG3, EGFR and ERBB2**. In the plot of -log_10_(*P*) v. map position, horizontal dashed lines are at *P *= 0.05, 0.01 and 0.001. Vertical dashed lines are at 20 kb intervals. The region covered by the gene is indicated in the centre of the figure by a horizontal line, with an arrow head indicating the direction of transcription. A crossing vertical line indicates the position of each exon.

The corresponding information for genes NRG2, NRG3, EGFR and ERBB2 is presented in Fig. 4, except that the LD maps are omitted for brevity. A single SNP in NRG2 showed significant genotypic association with schizophrenia (SNP RS2936651: map position 139401129 on Chr 5, OR = 2.081, CI 1.156–3.745). This finding was corroborated by a significant allelic test result also. Ten SNPs were significantly associated in NRG3, including one which was highly significantly associated (*P *< 0.01) by the allelic test (RS3924461: map position 084423371 on Chr10, OR = 1.283, CI 1.077–1.529). The pattern of LD in this gene (results not shown) is highly localised, similar to that in NRG1, so the observation of association from spatially separated SNPs may have arisen by chance or may suggest the presence of different functional variants within the same gene. The spatial pattern of association is not related to the distribution of the exons in this gene. Six SNPs in EGFR were significantly associated with schizophrenia, however, all fell in the range 0.01 <*P *< 0.05. Only four SNPs were studied in ERBB2, and none of these was found to be significantly associated with schizophrenia.

### Interaction analysis results

As summarised in Table 2, 79 SNPs were identified which had a *P*-value from an allelic or genotypic test that was less than 0.10 (details are given in Additional file 2). These SNPs were contained in the 6 genes considered above, namely NRG1, NRG2, NRG3, EGFR, ERBB2, and ERBB4. All pairs of these SNPs that are at least 0.5 cM apart on an interpolated Decode genetic map [21] were tested for interaction effects in a logistic regression interaction analysis.

A total of 2640 statistical tests for pair-wise interactions were performed (details of results are given in Additional file 2). Of these, 212 were significant at the 5% level: that is 8.03% of all tests were significant compared to 132 (= 5% of 2640) expected by chance if the null hypothesis of no interaction were true for all pairs of SNPs tested. If all the tests performed were mutually independent the probability that 212 or more would be significant by chance is 2.575 × 10^-11 ^(P(*R *≥ 212) where *R*~binomial(*N *= 2640, *p *= 0.05)). The true probability of such an outcome is certain to be larger, due to non-independence among the tests: nevertheless, this result provides convincing evidence that some of the observed interactions are real. The significance levels presented here are not adjusted for multiple testing: a Bonferroni correction would be inappropriate due to non-independence among the tests, and the multiple-testing issue is addressed by the comparison of observed and expected numbers of significant tests presented above. None of the significant results obtained would survive the Bonferroni correction.

Table 3 gives the percentage of significant interactions for each gene combination; the corresponding counts are given in brackets. There are four gene combinations in which the percentage of significant interactions is substantially higher than the 5% expected by chance, *viz*. the two-gene combinations NRG1-NRG2, NRG1-NRG3 and NRG2-EGFR, and the single large gene ERBB4. The percentages of significant interactions for combinations NRG1-NRG2 and NRG2-EGFR are based on a small number of tests and should therefore be treated with caution, but the percentages for NRG1-NRG3 and ERBB4 are based on substantial numbers.

**Table 3:** Percentages of pair-wise interactions that were significant (*P *< 0.05), classified by the gene(s) in which the interacting SNPs lie. Gene combinations in which there are substantially more significant interactions than the 5% expected by chance are indicated in bold. The counts that contribute to each percentage are also shown in parentheses as a fraction. The presence of "-" in the table indicates that no pairs of SNPs in this gene are 0.5 cM apart.

	**NRG1**	**NRG2**	**NRG3**	**EGFR**	**ERBB2**	**ERBB4**
**NRG1**	5.8% (6/103)	**13.6% (6/44)**	**11.7% (54/462)**	4.5% (7/154)	0.0% (0/22)	7.9% (45/572)
**NRG2**		-	4.8% (2/42)	**14.3% (2/14)**	0.0% (0/2)	7.7% (4/52)
**NRG3**			5.7% (6/105)	2.0% (3/147)	0.0% (0/21)	8.4% (46/546)
**EGFR**				-	0.0% (0/7)	5.5% (10/182)
**ERBB2**					-	7.7% (2/26)
**ERBB4**						**13.7% (19/139)**

**Figure 5:**
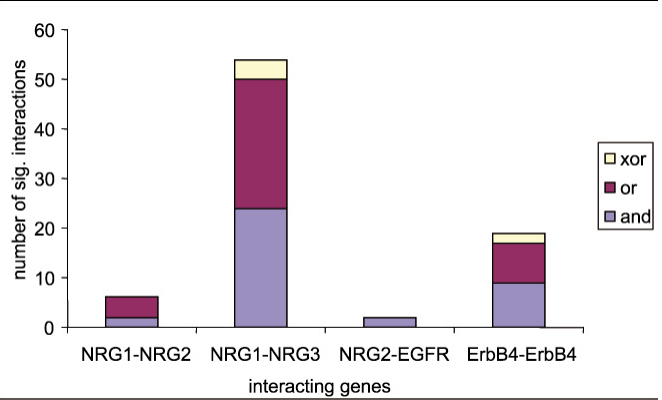
**Distribution of the types of pair-wise interaction in gene combinations with a large percentage of significant interactions**. The total in each column corresponds to the number of significant interactions presented in Table 3, i.e. the numerator of the fraction presented in that table for the gene combination in question.

The next step towards the identification of a mechanism to account for the statistical interactions is the identification of the type of each significant interaction. This was performed by pair-wise combination analysis (see Methods). Fig. 5 illustrates the distribution of the three types of interaction over the four gene combinations with a large percentage of significant interactions. The frequencies of *and-*type and *or-*type interactions are fairly similar. This might be expected, as the patterns of results that correspond to these two types of interaction are complementary, and equally likely to occur by chance. Interactions of the *xor*-type are rare: indeed, they are observed only in the two gene combinations that gave rise to a fairly large sample of significant interactions. This also might be expected, as it corresponds to a complex biological mechanism and to a rather specific pattern of results that is relatively unlikely to occur by chance.

## Discussion

In this manuscript we present confirmation of earlier published associations between schizophrenia and the genes NRG1 and ERBB4. We build a new, compelling picture of pathway involvement by showing that associations exist with additional gene family-members, namely EGFR, NRG2, and NRG3. Lastly, we present statistical evidence of gene epistasis, consistent with a highly complex biological pathway of schizophrenia susceptibility. This is the first time that the roles and inter-relationships of the ERBB and NRG gene families as a whole have been investigated in relation to schizophrenia and our approach sets a precedent in the investigation of common complex diseases.

Clear evidence for gene-gene interaction was detected for gene combinations NRG1-NRG2, NRG1-NRG3 and EGFR-NRG2, and suggestive evidence was also seen for ERBB4-NRG1, ERBB4-NRG2, ERBB4-NRG3 and ERBB4-ERBB2. Evidence of intragenic interaction was seen for SNPs in ERBB4. Of the 2640 tests for pair-wise interaction, 212 were significant at the 5% level, which is substantially more than the 131 expected by chance. This large number of low *P-*values may be partly explained by non-independence among tests. However, it may also indicate that some of the 79 SNPs studied interact to modulate susceptibility to schizophrenia.

Numerous studies have investigated the association between schizophrenia and NRG1 (reviewed in [22]). A comparison of the associations generated in the current study with those previously published shows good agreement. Associations that we observed at the 5' region of NRG1 overlap with those found by Stefansson et al. [3,4], Corvin et al. [23], Li et al. [24], Zhao et al. [25] and Petryshen et al. [26]. Similarly, the associations we observed at the 3' end of the gene overlap with those found by Yang et al. [27], Li et al. [23], Petryshen et al. [25] and Lachmann et al. [28]. Two polymorphisms in our study directly replicate these associations: the putatively functional SNP rs3924999 was found to be associated with schizophrenia in a Han Chinese family study conducted by Yang et al. [26]. This SNP was also tested in a Caucasian study of 136 families by Duan et al. [29], but was not significant. Rs2466049 was significant as part of a haplotype found in a Caucasian family by Petryshen et al. [25].

For the ERBB4 gene, four polymorphisms (rs3748962, rs839523, rs2054617 and rs6435711) screened in this study were also analysed by Silberberg et al. [8]. However, none of the individual SNP associations observed in the previous study was replicated here. As no underlying functional variant has been identified for ERBB4, this lack of association at the SNP level is unsurprising and is consistent with a heterogeneous haplotypic background across studies. It is worth noting also that non-coding polymorphisms might lead to dysregulation of ErbB4 expression levels, a hypothesis corroborated by the Silberberg et al. [8] study, which found enhanced expression of the ERBB4 Jm-a CYT-1 isoform (one of four splice variants, see [30]) in schizophrenic patients. The altered balance of ErbB4 isoforms could lead to variations in homo- and heterodimer formation and thus in downstream signalling pathways.

In addition to providing further evidence of NRG1 and ERBB4 associations to schizophrenia, our study is the first to suggest possible involvement of three additional genes from the NRG and ERBB gene families, i.e. NRG2, NRG3 and EGFR (ERBB1). It is interesting to note that we only detected evidence for association of NRG genes which are expressed in the central nervous system, namely NRG1, NRG2 and the nervous-system-specific NRG3 [31], whereas NRG4, which is mainly expressed in pancreas and absent from the brain [32] was not found to be associated to schizophrenia.

To our knowledge, an association of EGFR to schizophrenia has not previously been investigated. Association of a polymorphism in EGF (one of the six EGFR ligands, Fig. 1) with schizophrenia was reported in Finnish men [33], but not replicated in a Japanese population [34]. Further support for a possible role of EGF and EGFR in the disease comes from findings of abnormal expression of these genes in the forebrain and serum of schizophrenic patients [35]. EGFR is usually omitted in reviews on NRG-ERBB signalling, although it is well-established that ErbB4 can interact with and cross-phosphorylate EGFR upon neuregulin-binding [36-39]. Furthermore, EGFR and ERBB4 show partially overlapping temporal and spatial mRNA expression in several brain areas, such as cortex, striatum, cerebellum and hippocampus, and are coexpressed in GABAergic interneurons [40,41]. Therefore, it is very likely that EGFR/ErbB4 heterodimers mediate important functions in the developing and adult central nervous system *in vivo*. Whilst we limited this study to the NRG and ERBB gene families, it would be interesting to investigate association of the additional EGFR and ErbB4 ligands (see Fig. 1).

The significant gene-gene interactions reported in this study support our hypothesis of pathway involvement in schizophrenia. These genes have related functions and, for example, the NRG1-NRG2 and NRG1-NRG3 interactions may suggest competition between these ligands for a receptor. The NRG2-EGFR interaction is superficially more difficult to explain since NRG2 is a ligand for ErbB3 and ErbB4 and does not usually recognize EGFR directly or with high affinity [42-44]. However, a recent study reports that a single amino acid change in the EGFR ligand-binding domain dramatically increases the receptor affinity for NRG2β which can then induce potent stimulation of EGFR signalling [45]. The interactions within the ERBB4 gene suggest that combinations of particular genotypes may result in modified receptor activity, leading to altered risk of schizophrenia.

Such suggestions concerning possible mechanisms of interaction remain speculative until the reality of these interactions has been established biologically and the effect of each two-locus genotype on the phenotype is understood. A follow-up study currently underway, using independent cases and controls, will clarify which of these interactions can be replicated and may generate further hypotheses regarding the nature of the interactions. It will be interesting also to investigate whether observed associations or their interactions correspond to particular phenotypically defined patient sub-groups. The strong evidence of genetic associations and interactions reported here provides ample justification for such an enquiry.

## Conclusion

This study confirms previously published associations between schizophrenia and NRG1 and ERBB4, and points to three other members of the NRG and ERBB gene families (EGFR, NRG2 and NRG3) as potential schizophrenia susceptibility genes. Furthermore, the study presents evidence for inter- and intragenic pair-wise interaction between loci in genes of both families (NRG1-NRG2, NRG2-EGFR, NRG1-NRG3 and ERBB4-ERBB4). These extensive genetic inter-relationships observed among ligands and receptors of the ERBB and NRG gene families reflect the complexity of their interactions at the molecular level. The influence of so many closely related genetic factors on the potential development of disease pathophysiology has rarely, if ever, been demonstrated before and may partially explain the difficulty in arriving at an overarching genetic hypothesis of the etiology of schizophrenia. In future studies, it will be important to replicate our new findings in an independent cohort and to understand the underlying biological mechanisms of the single-locus associations as well as the interactions among different loci.

## Competing interests

The author(s) declare that they have no competing interests.

## Authors' contributions

IB helped to formulate the study and identified the genes of interest; she led the writing of the manuscript and provided the biological interpretation and context to our results.  AB led the statistical analysis, and produced the first draft of the manuscript.  BB performed the single-point statistical analyses and the pairwise analysis, and provided interpretation of his results.  NG provided interpretation of the linkage disequilibrium analysis and substantially contributed to the interpretation of the statistical analyses in the manuscript.  PM was instrumental in progressing the study, revised the manuscript and added important intellectual content concerning the biology of schizophrenia.  RM designed the statistical analysis plan and provided schizophrenia genetics expertise to the writing of the manuscript.  DS designed the laboratory aspects of the experiment and oversaw their execution.  DSC recruited, worked up clinically and supplied DNA from the Aberdeen schizophrenia case collection of 396 cases. PY provided the control collection of 1342 anonymous blood donors from Aberdeen. IP helped to formulate the original idea for the study and revised the manuscript critically, providing important intellectual content.  All authors read, edited and approved the final manuscript.

## Supplementary Material

Additional file 1 Detailed results from allelic and genotypic tests of single-point association of SNP loci with schizophrenia. Click here for file

Additional file 2 Detailed results from allelic and genotypic tests of association between SNP-pairs and susceptibility to schizophrenia. Click here for file
